# Incidence and Outcome of Acute Myocardial Infarction in Patients With Aortic Dissection and Risk Factor Control

**DOI:** 10.3389/fsurg.2021.678806

**Published:** 2021-09-09

**Authors:** Fang Liu, Si-Chong Qian, Shuai Jing, Zhe Wang, Xin-Chun Yang, Mu-Lei Chen

**Affiliations:** ^1^Department of Cardiology, Beijing Chaoyang Hospital, Capital Medical University, Beijing, China; ^2^Section of Cardiovascular Medicine, Yale University School of Medicine, New Haven, CT, United States; ^3^The Key Laboratory of Remodeling-Related Cardiovascular Disease, Department of Cardiology, Ministry of Education, Beijing Anzhen Hospital, Beijing Institute of Heart Lung and Blood Vessel Disease, Capital Medical University, Beijing, China; ^4^Department of General Surgery, Beijing Chaoyang Hospital, Capital Medical University, Beijing, China

**Keywords:** acute myocardial infarction, aorta dissection, incidence, long-term outcomes, risk factors

## Abstract

**Background and Aims:** The contradiction of management modality between acute myocardial infarction(AMI) and aortic dissection(AD) may result in clinical catastrophe. Data on risk factors, incidence, and outcome of AD and AMI are limited, and there have been no studies on the long-term outcomes of AMI in patients with AD. So we aimed to investigate long-term outcomes after AMI in patients with AD, and propose a useful diagnostic paradigm.

**Methods:** Consecutively enrolled patients with AD and AMI who were referred to our center from 2010 to 2017. Baseline patient characteristics, risk factors, all medical treatments, echocardiographic parameters, laboratory data, and treatment were recorded. All patients were followed up from the first hospitalization until a first heart event, death, or 17 March, 2018.

**Results:** 0.13% in AMI and 7.49% in AD patients had a concomitant diagnosis of AD and AMI. The average patient age was 53.3 ± 12.1 years and 84.6% were male. The most prevalent vascular risk factors were hypertension (69.2%) and current smoker (64.1%). Of all the 39 patients, 66.7% were managed surgically. Overall in-hospital mortality was 10.3%. The 30-day and 5-year fatality rates were 23.1% and 35.9%, but were higher for female than for male (66.7 vs. 30.3%, log-rank *P* = 0.045) on 5-year mortality. The overall survival of females was inferior to the males (log-rank *P* = 0.045).

**Conclusions:** Patients with AMI and AD exhibit high 5-year fatality rates. For these patients, surgical management tends to have lower mortality. Improved management of hypertension and smoking, may reduce future incidence rates.

## Background

Acute myocardial infarction (AMI) and aortic dissection (AD) are both very serious diseases with high rates of morbidity and mortality which have the same manifestations of chest pain. Acute aortic dissection (AAD) is the most common acute aortic condition requiring urgent surgical therapy, (1) with an incidence of about 3 cases in 100,000 per year (2, 3). Complications such as tamponade, aortic valve insufficiency, and malperfusion occur when the aortic side branches are involved (4). Thus, AAD represents a medical and/or surgical emergency (5). Presenting features are similar, and progression to dissection may occur (6–8). While the initiating event is unknown, most patients have a structural abnormality of the arterial wall and/or systemic hypertension (9, 10). Despite improved surgical techniques and perioperative care, 30-day and 5-year mortality remains high, between 15 and 30% (11, 12).

Many patients with AAD are diagnosed and treated as having acute coronary syndrome (ACS), which is a much more frequent condition than AAD (13). The triple rule-out protocol has been described as the one-stop computed tomography (CT) examination for chest pain designed to differentiate ACS, pulmonary embolism, and AAD (14). But there are a small proportion of patients with AD represent with AMI (approximately 1 ~3.6%) at the same time (15). However, clinical pathways of efficient streamlined care, similar to in ACS, have not yet been implemented (14). For cardiologists, prompt treatment with antiplatelet, antithrombotic, and thrombolytic agents is important once AMI is diagnosed. But the contradiction of management modality between AMI and acute aortic dissection may result in clinical catastrophe (16). Moreover, data on risk factors, incidence, and outcome of AD and AMI are limited, and there have been no studies on the long-term outcomes of AMI in patients with AD. This study, therefore, aimed to investigate long-term outcomes after AMI in patients with AD, and propose a useful diagnostic paradigm.

## Methods

### Study Population and Baseline Characteristics

All patients (age ≥18 years) with AD and AMI who were referred to Anzhen Hospital in Beijing, China, from January 2010 and December 2017, were consecutively enrolled in this study. Aortic dissection was classified according to the Stanford system into type A (involvingg the ascending aorta protximal to left subclavian artery origin) or type B (involving only the descending aorta) (17). AD diagnosis was based on history, imaging study findings, visualization at surgery, and/or postmortem examination (1). AMI was diagnosed if a patient had a cardiac troponin I level >99th percentile with ≥1 of the following: chest pain lasting >20 min or diagnostic serial electrocardiographic changes consisting of new pathological Q waves, new ST-segment-T-wave changes, or new left bundle branch block (18). The exclusion criteria included rheumatic heart disease, severe congestive heart disease, malignant tumor, and use of the oral contraceptive pill or pregnancy. All participants provided written informed consent.

Baseline patient characteristics were recorded from the patients, their hospital records, and their general practice records, details of the clinical event, medication, past medical history, all investigations relevant to their admission, and all interventions occurring subsequent after the event. Standardized clinical history and cardiovascular examination were recorded. Analyzed risk factors included age, sex, body mass index (BMI), current smoker, hypertension, diabetes, and hyperlipidemia. Clinically relevant comorbidities included cardiac failure, atrial fibrillation, known coronary artery disease, prior aortic dissection, prior myocardial infarction, anterior myocardial infarction, known aortic aneurysm, prior stroke/ transient ischemic attack (TIA), carotid artery disease, peripheral artery disease chronic renal insufficiency and prior cardiac surgery. All medical treatments during hospitalization were recorded, including aspirin, clopidogrel, statin, nitrates, diuretic, warfarin, and antihypertensives.

Echocardiographic parameters were assessed using transthoracic echocardiography with the Teichholz method before coronary angiography (19). Parameters analyzed included pericardial effusion, acute aortic valve insufficiency, left ventricular ejection fraction (LVEF), and ascending aortic diameters.

Laboratory data were collected upon admission to the hospital, including levels of total cholesterol (TC), triglycerides (TG), high-density lipoprotein cholesterol (HDL-C), low-density lipoprotein cholesterol (LDL-C), creatine kinase-MB (CK-MB), troponin I (TNI), D-Dimer, homocysteine (Hcy) and high-sensitivity C-reactive protein (hs-CRP).

Coronary angiography was performed according to standard criteria. Offline analysis of digital angiograms was performed in the core laboratory using an automated edge detection system (CMS; Medis Medical Imaging Systems, Leiden, the Netherlands). Binary stenosis was defined as stenosis of >50% of the luminal diameter.

### Follow-Up and Definition of Endpoints

The follow-up started on the day of admission. In the hospital, major adverse events recorded included death, cardiogenic shock, ventricular tachyarrhythmia (VT), ventricular fibrillation (VF), acute left-sided heart failure, and acute kidney injury (AKI).

After hospital discharge, adverse events including cardiac death, recurrent myocardial infarction (re-MI), hospital re-admission and recurrent angina. If a recurrent vascular event was suspected, the patient was assessed by a clinical research physician. Event rates were defined as the total number of vascular events that led to different clinical presentations during the study period. An extension of a previous dissection was not considered to be a recurrent event if it occurred within 6 months of the first event.

All patients were followed up from the first hospitalization until a first coronary event, death, or 17 March, 2018. Endpoint status was ascertained via clinic visits, medical records, telephone contact, and text messages. For deceased patients, death certificates were obtained, and the next of kin were interviewed to determine when the death occurred. This study conformed to the principles defined in the Declaration of Helsinki. Local ethical committee approval was obtained. All patients provided their informed consent prior to their inclusion in the study.

### Statistical Analysis

Continuous variables with normal distributions were expressed as mean ± standard deviation, and compared using one-way ANOVA analysis of variance or Fisher's exact test. Categorical variables were expressed as frequencies with percentages, and compared using the chi-square test where appropriate. The 95% confidence interval (CI) of annual mortality rate was calculated using the binomial approximation. Survival was graphically represented using Kaplan-Meier curves. Differences in survival rates were compared using the log-rank test. Univariate and multivariate Cox proportional hazards models were used to identify study endpoint predictors. Variables with univariate *p* values < 0.10 were selected for multivariate analysis and expressed as hazard ratios (HRs) with 95% CIs. Multivariate Cox regression analysis was performed using an enter method. The 95% CI of the annual mortality rate was calculated using GraphPad Prism 7 (GraphPad Software Inc., La Jolla, USA). All other analyses were performed using SPSS statistical software, version 25.0 (SPSS Inc., Chicago, USA). All tests were 2-tailed, and statistical significance was defined as *p* < 0.05.

## Results

### Demographics and Risk Factors

**Figure 1:**
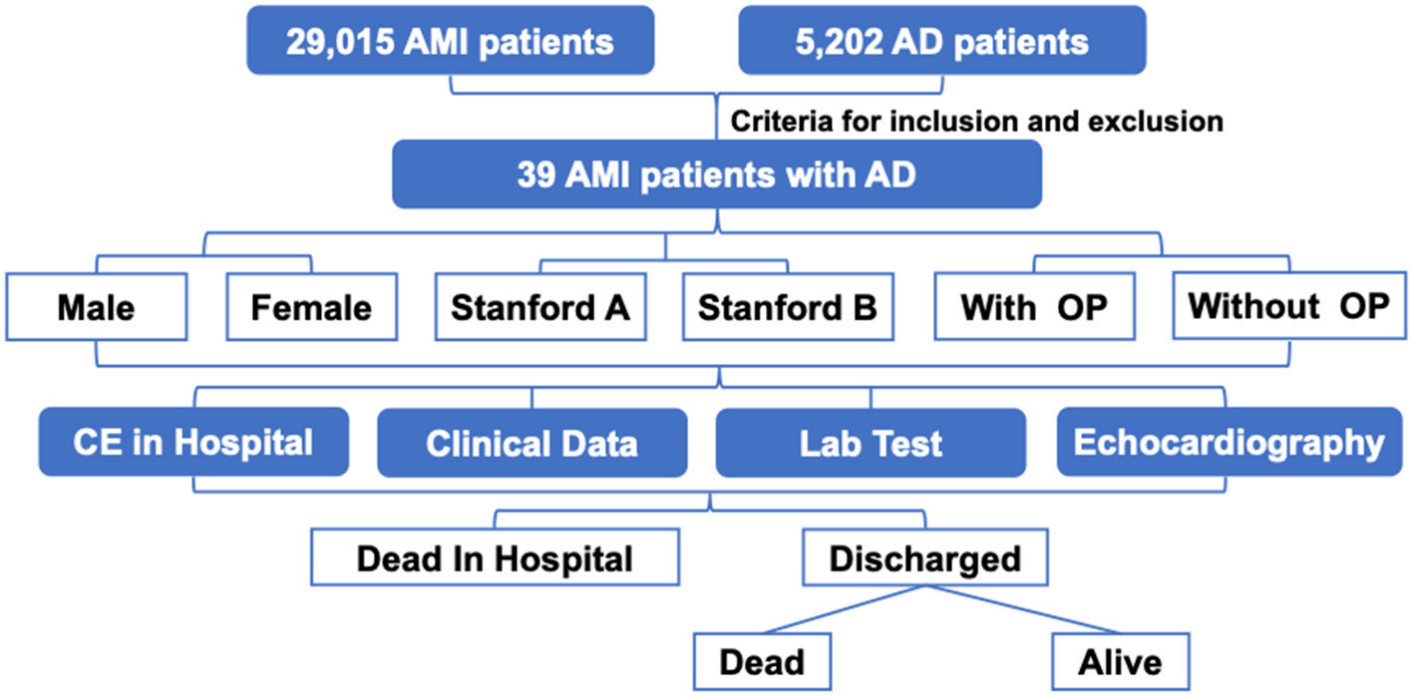
The protocol of this study. There were 39 patients with AD and AMI among 29,015 patients who were hospitalized with AMI and 5,202 patients with AD; All of them completed the follow-up. AD indicates aortic dissection; AMI, acute myocardial infarction; CE, cardiac events.

From 2010 to 2017, 29,015 patients with AMI and 5,202 patients with AD were identified who were hospitalized in Anzhen Hospital. Of these, 39 AMI patients had a concomitant diagnosis of AD (0.13% in AMI vs. 7.49% in AD) (Figure 1). Table 1 shows the demographics and risk factors for AMI patients with AD by sex and by Stanford classification. The average patient age was 53.3 ± 12.1 years and 84.6% were male. Of the 39 patients, 31 (79.5%) were Stanford type A and 8 (20.5%) were Stanford type B. 6 patients had previous aorta dissection during the study period (3 of them are Stanford type A, 3 are Stanford type B); 11 patients had prior MI (9 are Stanford type A, 2 are Stanford type B). The most prevalent vascular risk factors were hypertension (69.2%) and current smoker (64.1%). Hypertension tended to be more common in Type B patients (*P* = 0.042, Table 1). There were no statistically significant sex differences in risk factors for AMI patients with AD, although men were more likely to be on triple antihypertensive medication (*p* = 0.040, Table 1). Compared with Type B patients with AMI, Type A patients were less likely to present with ST-elevation myocardial infarction (45.2 vs. 87.5%, *P* = 0.049) (Table 1). A history of cardiac surgery was present in 14 patients (35.9%). In the hospital, Type A patients had lower rates of medication use compared to Type B patients, including statin (32.3 vs. 75.0%, *P* = 0.045), aspirin (38.7 vs. 87.5%, *P* = 0.020), clopidogrel (22.6 vs. 75.0%, *P* = 0.010) and nitrates (38.7 vs. 87.5%, *P* = 0.020) (Table 1). 18 (58.1%) of Type A patients had pericardial effusion and 19 (61.3%) of them had acute aortic valve insufficiency, the mean ascending aortic diameter was 46.2 ± 12.1 mm in the Type A group with a range from 24.3 to 69.6 mm. Hcy in male is 1.25-fold of that in female (14.8 ± 2.9 vs. 11.8 ± 1.9, *P* = 0.021), and higher in type B dissection patients compared with type A group (17.8 ± 3.5 vs. 13.4 ± 2.0, *P* < 0.001).

**Table 1:** Demographics and risk factors for incident aortic dissection by sex and type.

	**Total**	**Male**	**Female**	***P* Value**	**Type A**	**Type B**	***P* Value**
	***n* = 39**	***n* = 33**	***n* = 6**		***n* = 31**	***n* = B**	
Mean (SD) age, y	53.3 ± 12.1	51.9 ± 11.9	60.8 ± 11.3	0.098	52.0 ± 11.4	58.4 ± 14.1	0.188
Male, *n* (%)	33 (84.6)				25 (80.6)	8 (100.0)	0.313
BMI, *n* (%)	25.5 ± 3.3	25.5 ± 3.2	25.4 ± 3.9	0.920	25.1± 3.5	27.2 ± 1.8	0.096
KILLIP≥2, *n* (%)	26 (66.7)	21 (63.6)	5 (83.3)	0.643	20 (64.5)	6 (75.0)	0.694
**Risk factors**							
Current smoker, *n* (%)	25 (64.1)	21 (63.6)	4 (66.7)	1.000	19 (61.3)	6 (75.0)	0.686
Hypertension, *n* (%)	27 (69.2)	23 (69.7)	4 (66.7)	1.000	19 (61.3)	8 (100.0)	0.042^*^
Diabetes mellitus, *n* (%)	6 (15.4)	6 (18.2)	0 (0.0)	0.564	3 (9.7)	3 (37.5)	0.088
Dyslipidemia, *n* (%)	8 (20.5)	6 (18.2)	2 (33.3)	0.583	6 (19.4)	2 (25.0)	0.658
Cardiac failure, *n* (%)	13 (33.3)	10 (33.3)	3 (50.0)	0.380	8 (25.8)	5 (62.5)	0.090
Atrial fibrillation, *n* (%)	3 (7.7)	2 (6.1)	1 (16.7)	0.403	2 (6.5)	1 (12.5)	0.508
**AMI characteristics**							
Anterior infarction, *n* (%)	16 (41.0)	13 (39.4)	3 (50.0)	0.674	11 (35.5)	5 (62.5)	0.235
Inferior infarction, *n* (%)	26 (66.7)	22 (66.7)	4 (66.7)	1.000	21 (67.5)	5 (62.5)	1.000
ST-segment elevation myocardial infarction, *n* (%)	21 (53.8)	20 (60.6)	1 (16.7)	0.077	14 (45.2)	7 (87.5)	0.049^*^
**Previous vascular disease**							
Known coronary artery disease, *n* (%)	13 (33.3)	10 (33.3)	3 (50.0)	0.380	12 (38.7)	1 (12.5)	0.229
Prior aortic dissection, *n* (%)	6 (15.4)	5 (15.2)	1 (16.7)	1.000	3 (9.7)	3 (37.5)	0.088
Prior myocardial infarction, *n* (%)	11 (28.2)	8 (24.2)	3 (50.0)	0.323	9 (29.0)	2 (25.0)	1.000
Known aortic aneurysm, *n* (%)	23 (59.0)	20 (60.6)	3 (50.0)	0.674	20 (64.5)	3 (37.5)	0.235
Stroke, *n* (%)	5 (12.8)	4 (12.1)	1 (16.7)	1.000	4 (12.9)	1 (12.5)	1.000
Carotid artery disease, *n* (%)	2 (5.1)	2 (6.1)	0 (0.0)	1.000	1 (3.2)	1 (12.5)	0.372
Peripheral arterial disease, *n* (%)	1 (2.6)	1 (3.0)	0 (0.0)	1.000	1 (3.2)	0 (0.0)	1.000
Chronic renal insufficiency, *n* (%)	3 (7.7)	2 (6.1)	1 (16.7)	0.403	2 (6.5)	1 (12.5)	0.508
Prior cardiac surgery, *n* (%)	14 (35.9)	11 (33.3)	3 (50.0)	0.647	10 (32.3)	4 (50.0)	0.424
Aortic valve replacement, *n* (%)	3 (7.7)	2 (6.1)	1 (16.7)	0.403	3 (9.7)	0 (0.0)	1.000
Descending aorta replacement, *n* (%)	4 (10.3)	4 (12.1)	0 (0.0)	1.000	1 (3.2)	3 (37.5)	0.022^*^
Coronary artery bypass graft surgery, *n* (%)	1 (2.6)	0 (0.0)	1 (16.7)	0.154	1 (3.2)	0 (0.0)	1.000
Percutaneous Coronary Intervention, *n* (%)	5 (12.8)	4 (12.1)	1 (16.7)	1.000	4 (12.9)	1 (12.5)	1.000
Aortic root surgery, *n* (%)	2 (5.1)	1 (3.0)	1 (16.7)	0.287	2 (6.5)	0 (0.0)	1.000
**Medications**							
Statin, *n* (%)	16 (41.0)	13 (39.4)	3 (50.0)	0.674	10 (32.3)	6 (75.0)	0.045^*^
Aspirin, *n* (%)	19 (48.7)	17 (51.5)	2 (33.3)	0.661	12 (38.7)	7 (87.5)	0.020^*^
Clopidogrel, *n* (%)	13 (33.3)	10 (33.3)	3 (50.0)	0.380	7 (22.6)	6 (75.0)	0.010^*^
Nitrates, *n* (%)	19 (48.7)	17 (51.5)	2 (33.3)	0.661	12 (38.7)	7 (87.5)	0.020^*^
Diuretic, *n* (%)	21 (53.8)	20 (60.6)	1 (16.7)	0.077	16 (51.6)	5 (62.5)	0.702
Warfarin, *n* (%)	11 (28.2)	9 (27.3)	2 (33.3)	1.000	11 (35.5)	0 (0.0)	0.078
Antihypertensives				0.040^*^			0.201
0, *n* (%)	8 (20.5)	8 (24.2)	0 (0.0)		8 (25.8)	0 (0.0)	
1, *n* (%)	9 (23.1)	6 (18.2)	3 (50.0)		8 (25.8)	1 (12.5)	
2, *n* (%)	10 (25.6)	7 (21.2)	3 (50.0)		6 (19.4)	5 (50.0)	
≥3, *n* (%)	12 (30.8)	12 (36.4)	0 (0.0)		9 (29.0)	3 (37.5)	
**Echocardiography**							
Pericardial effusion, *n* (%)	19 (48.7)	16 (48.5)	3 (50.0)	1.000	18 (58.1)	1 (12.5)	0.044^*^
Acute aortic valve insufficiency, *n* (%)	21 (53.8)	18 (54.5)	3 (50.0)	1.000	19 (61.3)	2 (25.0)	0.112
LVEF ≤ 40, *n* (%)	7 (17.9)	6 (18.2)	1 (16.7)	1.000	4 (12.9)	3 (37.5)	0.137
Ascending aortic diameters (*n* = 33)	44.4 ± 11.4	44.7 ± 11.3	42.7 ± 12.6	0.695	46.2 ± 12.1	37.5 ± 3.0	0.001^*^
**Biochemical**							
TC, mmol/L	3.9 ± 1.0	4.0 ± 1.1	3.5 ± 0.6	0.461	4.0 ± 1.0	3.7 ± 1.2	0.535
TG, mmol/L	1.6 ± 1.0	1.5 ± 1.1	1.7 ± 0.3	0.709	1.6 ± 1.0	1.6 ± 0.9	0.852
HDL, mmol/L	1.0 ± 0.6	1.0 ± 0.7	1.0 ± 0.2	0.985	1.0 ± 0.7	0.8 ± 0.3	0.293
LDL, mmol/L	2.3 ± 0.9	2.4 ± 0.9	1.9 ± 0.4	0.262	2.3 ± 0.8	2.3 ± 1.0	0.944
CK-MB, ng/mL	150.4 ± 98.6	141.3 ± 101.1	222.8 ± 17.8	0.284	133.3 ± 99.5	210.0 ± 78.0	0.177
TNI, ng/mL	46.0 ± 30.7	42.9 ± 30.0	74.6 ± 27.4	0.171	40.2 ± 29.8	69.2 ± 25.4	0.092
D-DIMER, mg/L	1964.8 ± 2720.2	2209.2 ± 2833.5	259.3 ± 406.6	0.256	2276.6 ± 3004.4	796.6 ± 439.7	0.292
HCY, umol/L	14.3 ± 3.0	14.8 ± 2.9	11.8 ± 1.9	0.021^*^	13.4 ± 2.0	17.8 ± 3.5	0.000^*^
hs-CRP, mg/L	18.3 ± 14.1	19.1 ± 14.0	13.2 ± 16.3	0.536	18.7 ± 13.5	17.0 ± 16.6	0.773

*AMI, acute myocardial infarction; BMI, body mass index; CK-MB, creatine kinase isoenzymes; HCY, homocysteine; HDL, high-density lipoprotein cholesterol; hs-CRP, hypersensitive-c reactive protein; LDL, low-density lipoprotein; LVEF, left ventricular ejection fraction; TC, total cholesterol; TG, triglycerides; TNI, troponin I*.

* *P < 0.05*.

### Presenting Signs, Symptoms and Diagnostic Imaging

Hypertension at initial presentation was more common among patients with Type B dissection (61.3 vs. 100%, *P* = 0.042). Despite 79.5% of patients being on antihypertensive medication, the control of blood pressure (BP) was poor. Maximum previously recorded systolic BP was ≥180 mmHg (Stage 3) in 51.9% of patients and was similar for type B dissections (52.6%) and type A dissections (50.0%), but tended to be more common in women (75.0%) in comparison with men (47.8%) (Table 2).

**Table 2:** Presenting symptoms and physical examination of patients with acute myocardial infarction and aortic dissection.

	**Total**	**Male**	**Female**	***P* Value**	**Type A**	**Type B**	***P* Value**
	***n* = 39**	***n* = 33**	***n* = 6**		***n* = 31**	***n* = B**	
Hypertension, *n* (%)	27 (69.2)	23 (69.7)	4 (66.7)	1.000	19 (61.3)	8 (100.0)	0.042^*^
Control under 140/90 mmHg, *n* (%)	17 (43.6)	15 (45.5)	2 (33.3)	0.679	15 (48.4)	2 (25.0)	0.426
Degree				0.782			1.000
Stage 1, *n* (%)	7 (25.9)	6 (26.1)	1 (25.0)		5 (26.3)	2 (25.0)	
Stage 2, *n* (%)	6 (22.2)	6 (26.1)	0 (0.0)		4 (21.1)	2 (25.0)	
Stage 3, *n* (%)	14 (51.9)	11 (47.8)	3 (75.0)		10 (52.6)	4 (50.0)	
Presenting symptoms				0.514			0.658
Chest pain, *n* (%)	31 (79.5)	26 (78.8)	5 (83.3)		25 (80.6)	6 (75.0)	
Feelings of pressure or tightness, *n* (%)	5 (12.8)	5 (15.2)	0 (0.0)		4 (12.9)	1 (12.5)	
Pain throat/jaw, *n* (%)	2 (5.1)	1 (3.0)	1 (16.7)		1 (3.2)	1 (12.5)	
Syncope, *n* (%)	1 (2.6)	1 (3.0)	0 (0.0)		1 (3.2)	0 (0.0)	
Auscultated murmur of aortic insufficiency, *n* (%)	12 (30.8)	11 (33.3)	1 (16.7)	0.645	11 (35.5)	1 (12.5)	0.394
Diagnostic imaging of AD				0.467			0.058
Computed tomography, *n* (%)	21 (53.8)	19 (57.6)	2 (33.3)		15 (48.4)	6 (75.0)	
Echocardiophy, *n* (%)	11 (28.2)	8 (24.2)	3 (50.0)		11 (35.5)	0 (0.0)	
Magnetic resonance imaging, *n* (%)	3 (7.7)	2 (6.1)	1 (16.7)		1 (3.2)	2 (25.0)	
Coronary angiography, *n* (%)	3 (7.7)	3 (9.1)	0 (0.0)		3 (9.7)	0 (0.0)	

*AD, aortic dissection*.

* *P < 0.05*.

The most frequent presentation is sudden-onset chest pain (79.5%). 17.9% of patients presented with feelings of pressure or tightness or throat/jaw pain (Table 2), which were more similar to symptoms of AMI. Not infrequently, 1 male patients with type A dissection presented with syncope and transient blindness without other neurological findings, 5 type A patients presented with paraparesis or paraplegia.

Most patients had multiple imaging studies performed (Table 2). CT was more often the initial study tool for 53.8% of all the patient, particularly in patients with type B dissection. Echocardiography and magnetic resonance imaging (MRI) were rarely used initially. 3 male patients with type A dissection were not diagnosed until coronary angiography was done.

### Angiographic Features, and Treatment Characteristics

Of all the 39 patients, 66.7% (26/39) were managed surgically (Table 3), 16 of them were treated AMI and AD at the same time.

**Table 3:** Angiographic features, and treatment characteristics.

	**Type A**	**Type B**	***P* Value**
	***n* = 31**	***n* = 8**	
**Surgical Management**, *n* (%)	20 (64.5)	6 (75.0)	0.694
On AMI, *n* (%)	19 (61.3)	6 (75.0)	0.686
On AD, *n* (%)	17 (54.8)	4 (50.0)	1.000
On AMI and AD, *n* (%)	12 (38.7)	4 (50.0)	0.425
**AMI Treatment strategy**			
Angiographic features			
LAD, *n* (%)	9 (29.0)	8 (100.0)	0.000^*^
LCx, *n* (%)	5 (16.1)	5 (62.5)	0.016^*^
RCA, *n* (%)	22 (71.0)	5 (62.5)	0.682
Involvement of the LMCA, *n* (%)	1 (3.2)	0 (0.0)	1.000
Vascular involvement number			0.007^*^
Nonstenotic vessels, *n* (%)	5 (16.1)	0 (0.0)	
1-vessel disease, *n* (%)	18 (58.1)	2 (25.0)	
2-vessel disease, *n* (%)	6 (19.4)	1 (12.5)	
3-vessel disease, *n* (%)	2 (6.5)	5 (62.5)	
Treatment strategy			
Thrombolysis, *n* (%)	2 (6.5)	0 (0.0)	1.000
Percutaneous coronary intervention, *n* (%)	6 (19.4)	1 (12.5)	1.000
Coronary artery bypass grafting, *n* (%)	13 (41.9)	4 (50.0)	0.709
No revascularization, *n* (%)	11 (35.5)	3 (37.5)	1.000
**AD Treatment strategy**	*n =* 16	*n =* 2	
Procedural times(min)			
Cardiopulmonary bypass	209 ± 93	451 ± 30	0.003^*^
Cross-clamp	118 ± 57	149 ± 45	0.004^*^
Selective cerebral perfusion	25.0 ± 5.7	41.0 ± 5.7	0.467

*AD, aortic dissection; AMI, acute myocardial infarction; LAD, left anterior descending; LCx, left circumflex; LMCA, left main coronary artery; RCA, right coronary artery*.

* *P < 0.05*.

The vascular involvement number was significantly different between patients with type A dissection and patients with type B dissection (*P* = 0.007); patients with type A dissection were more likely to have one vessel involvement than patients with type B dissection (Table 3). Left anterior descending (LAD) and left circumflex (LCx) were more often involved in patients with type B dissection (LAD: 100.0 vs. 29.0%, LCx: 62.5 vs. 16.1%, *P* < 0.05) (Table 3), however, not all these coronary vascular involvements that need surgical intervention, and the ratio of percutaneous coronary intervention were similar between type A group (19.4%, 6/31) and type B group (12.5%, 1/8, *P* = 1.000), in addition, the ratio of coronary artery bypass grafting performed in type A patients was no significance different compared with type B patients (41.9%, 13/31 vs. 50%, 4/8, *P* = 0.709).

A total of 18 open procedures were performed following acute dissection during the study period. Both the cardiopulmonary bypass time (209 ± 93 vs. 451 ± 30) and cross-clamp time (118 ± 57 vs. 149 ± 45) are less in type A dissection group compared with type B group (*P* < 0.05), however, there is no significant difference in selective cerebral perfusion time between two groups.

### In-Hospital Management and Follow-Up

**Figure 2:**
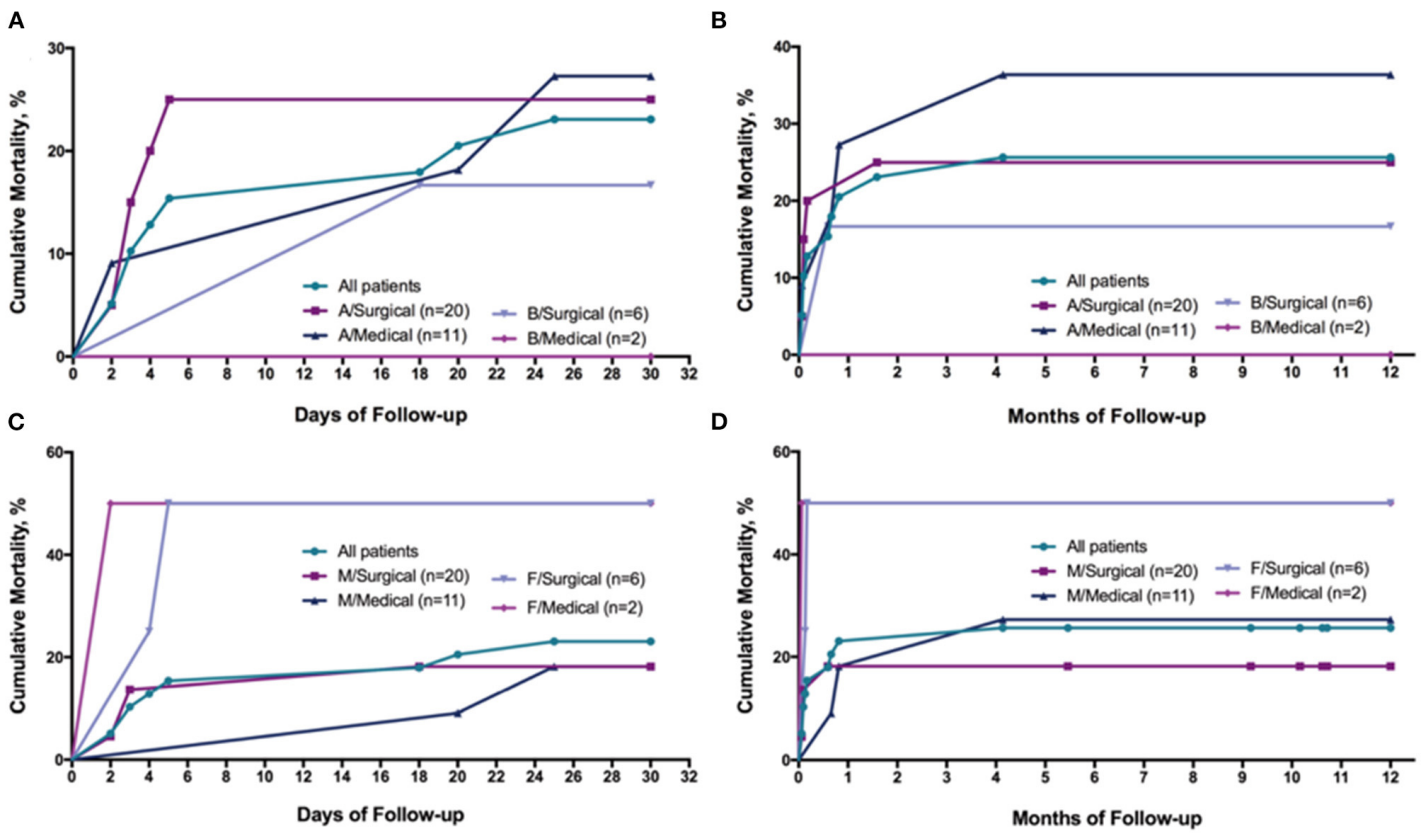
Cumulative Mortality curves for clinical outcomes in the 39 patients with AD with AMI. **(A)**, Cumulative Mortality curves for 30 days between Type A and Type B; **(B)**, Cumulative Mortality curves for 12 months between Type A and Type B; **(C)**, Cumulative Mortality curves for 30 days between men and women; **(D)**, Cumulative Mortality curves for 12 months between men and women; AD indicates aortic dissection; AMI, acute myocardial infarction.

**Figure 3:**
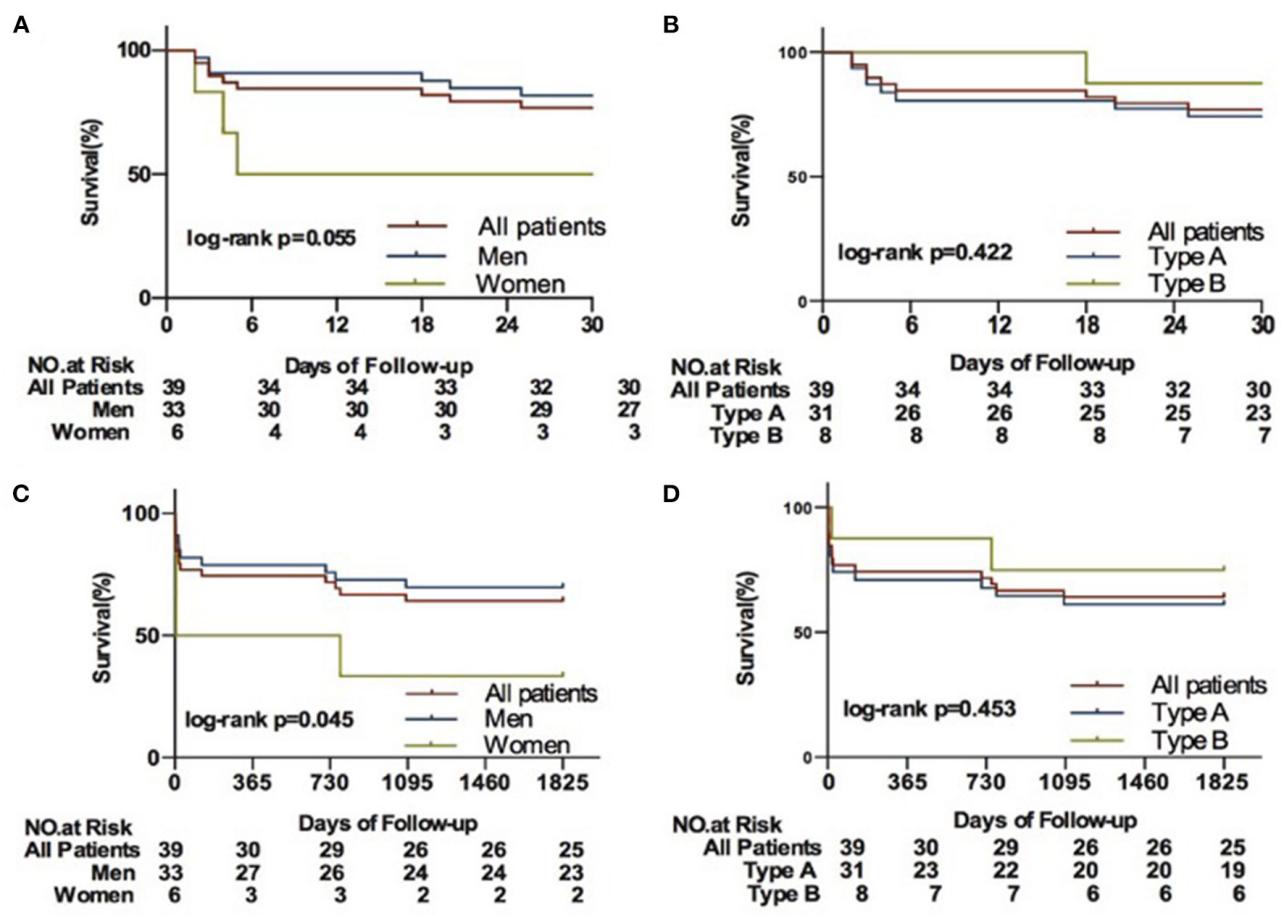
Kaplan-Meier curves for clinical outcomes in the 39 patients with AD with AMI. **(A)**, Kaplan-Meier curves for 30 days between men and women; **(B)**, Kaplan-Meier curves for 30 days between Type A and Type B; **(C)**, Kaplan-Meier curves for 5 years between men and women; **(D)**, Kaplan-Meier curves for 5 years between Type A and Type B; AD indicates aortic dissection; AMI, acute myocardial infarction.

Overall in-hospital mortality was 10.3%. Death all occurred in patients receiving surgery (10.9%). For patients surviving until hospital discharge, the median length of stay was 15.3 ± 11.8 days. Follow-up at 3.02 ± 2.68 years was available for all of the patients, 14 died during the study period. Mortality was highest within the first 7 days of presentation (Figure 2). The 30-day and 5-year fatality rates (Figure 3) were 23.1% and 35.9%, but were higher for female than for male (66.7 vs. 30.3%, log-rank *P* = 0.045) on 5-year mortality. In patients with incident type A dissection who survived while hospital admission, 30-day mortality was 25.8%. Among those who survived while hospital discharge, subsequent 5-year survival rates were high (61.3% for type A; 75% for type B). After reported, 42.9% of death was caused by cardiac, 28.6% was caused by aortic rupture (Table 4).

**Table 4:** In- and out-hospital outcomes.

	**Total**	**Male**	**Female**	**P Value**	**Type A**	**Type B**	**P Value**
	***n =* 39**	***n =* 33**	***n =* 6**		***n =* 31**	***n =* 8**	
**In-hospital outcome**							
Death, *n* (%)	4 (10.3)	2 (6.1)	2 (33.3)	0.104	3 (9.7)	1 (12.5)	1.000
Complications							
Cardiogenic shock, *n* (%)	6 (15.4)	5 (15.2)	1 (16.7)	1.000	4 (12.9)	2 (25.0)	0.583
VTA, *n* (%)	4 (10.3)	4 (12.1)	0 (0.0)	1.000	2 (6.5)	2 (25.0)	0.180
VF, *n* (%)	3 (7.7)	2 (6.1)	1 (16.7)	0.403	2 (6.5)	1 (12.5)	0.508
Acute left-sided heart failure, *n* (%)	9 (23.1)	8 (24.2)	1 (16.7)	1.000	6 (19.4)	3 (37.5)	0.355
AKI, *n* (%)	4 (10.3)	4 (12.1)	0 (0.0)	1.000	3 (9.7)	1 (12.5)	1.000
Length of stay, d	15.3 ± 11.8	16.2 ± 11.6	10.8 ± 12.9	0.316	13.9 ± 11.0	20.9 ± 14.0	0.138
**Out-hospital outcome**							
Follow-up,y	3.02 ± 2.68	3.3 ± 2.7	1.5 ± 1.8	0.129	2.8 ± 2.7	3.9 ± 2.7	0.336
Death, *n* (%)	14 (35.9)	10 (33.3)	4 (66.7)	0.163	12 (38.7)	2 (25.0)	0.686
Aortic rupture, *n* (%)	4 (28.6)	2 (20.0)	2 (50.0)	0.104	4 (33.3)	0 (0.0)	1.000
Cardiac death, *n* (%)	6 (42.9)	4 (12.1)	2 (50.0)	0.224	3 (33.3)	2 (100.0)	0.583
MI, *n* (%)	3 (7.7)	2 (6.1)	1 (16.7)	0.403	2 (6.5)	1 (12.5)	0.508
Re-hospital, *n* (%)	3 (7.7)	2 (6.1)	1 (16.7)	0.403	1 (3.2)	2 (25.0)	0.101
Recurrent angina, *n* (%)	2 (5.1)	2 (6.1)	0 (0.0)	1.000	0 (0.0)	2 (25.0)	0.038^*^

*AKI, acute kidney injury; MI, myocardial infarction; VF, ventricular fibrillation; VT, ventricular tachyarrhythmia*.

* *P < 0.05*.

Female patients tended to be older (60.8 vs. 51.9, *P* =0.098) (Table 1) and the overall survival of females was inferior to the males (log-rank *P* = 0.045; Figure 3). However, it seems that there is no specific difference in mortality between type A and type B dissection (*P* > 0.05).

Of 31 patients with type A dissection, 20 (64.5%) were managed surgically (Supplementary Table 1). The overall surgical in-hospital mortality was 15.0%; medically treated patients had an in-hospital mortality of 0%. Surgery was not performed in 35.5% of patients with type A dissection because of advanced age, comorbidity, patient refusal, intramural hematoma, and death before planned surgery. Of type B patients, 6 were managed surgically resulting in 1 death (16.7%), 2 were managed medically with 0 death (0%). Patients treated medically had lower mortality in-hospital but as for the long-term outcome, surgical management tends to have lower mortality. Among type A patients, 20 received surgical therapy, of whom 6 death (out-hospital mortality 30.0%), and 11 received medical therapy, of whom 6 died (54.5%); Of type B patients, 1 death (our-hospital mortality 16.7%) occurred after surgery and 1 death (50.0%) happened with medical management.

Cox proportional hazards models for all-cause mortality are shown in Table 5. By sex, the independent determinants of deaths were aortic dissection type (HR: 30.432, 95% CI: 1.092–848.183), prior cardiac surgery (HR: 0.048, 95% CI: 0.003–0.745), auscultated murmur of aortic insufficiency (HR: 18.258, 95% CI: 1.436–232.18), and medications such as nitrates (HR: 0.045, 95% CI: 0.003–0.578) and warfarin (HR: 0.033, 95% CI: 0.001–0.915). As for by type, the independent determinants of deaths were male (HR: 0.001, 95% CI: 9.296–5355.344), prior cardiac surgery (HR: 0.004, 95% CI: 0.001–0.266), and medications such as antihypertensives (HR: 0.036, 95% CI: 0.074–0.915) and warfarin (HR: 0.020, 95% CI: 0.001–0.528).

**Table 5:** Cox regression analysis for death over the follow-up.

	**Univariate**		**Multivariate**	
	**HR (95%CI)**	***P* Value**	**HR (95%CI)**	***P* Value**
**By sex**				
Aortic dissection type	0.727 (0.154–3.424)	0.687	30.432 (1.092–848.183)	0.044
Prior cardiac surgery	0.097 (0.012–0.774)	0.028	0.048 (0.003–0.745)	0.030
Auscultated murmur of aortic insufficiency	2.999 (1.040–8.647)	0.042	18.258 (1.436–232.18)	0.025
On AD	0.301 (0.093–0.973)	0.045		
On AMI and AD	0.289 (0.080–1.047)	0.059		
Antihypertensives	0.398 (0.212–0.746)	0.004		
Nitrates	0.243 (0.065–0.902)	0.035	0.045 (0.003–0.578)	0.017
Warfarin	0.125 (0.016–0.977)	0.047	0.033 (0.001–0.915)	0.033
**By type**				
Male	2.814 (0.841–9.414)	0.093	223.123 (9.296–5355.344)	0.001
KILLIP≥2	3.891 (0.866–17.472)	0.076		
Inferior infarction	2.803 (0.945–8.310)	0.063		
Prior cardiac surgury	0.241 (0.054–1.086)	0.064	0.015 (0.001–0.266)	0.004
Auscultated murmur of aortic insufficiency	2.678 (0.886–8.100)	0.081		
On AD	0.266 (0.083–0.859)	0.027		
On AMI and AD	0.306 (0.084–1.106)	0.071		
Antihypertensives	0.431 (0.237–0.783)	0.006	0.261 (0.074–0.915)	0.036
Aspirin	0.316 (0.081–1.231)	0.097		
Nitrates	0.180 (0.037–0.889)	0.035		
Warfarin	0.117 (0.015–0.908)	0.040	0.018 (0.001–0.528)	0.020

*AD, aortic dissection; AMI, acute myocardial infarction*.

## Discussion

Patients suffered from both AMI and AD may be uncommon, but complications occur often and early, and the outcome is frequently fatal. Although clinicians today are better equipped to deal with the complex threat posed by AMI and AD, mortality rates remain high. Besides that, there has not been any research focus on the long-term outcome of AMI patients with AD. The results of our study demonstrated that patients with AMI and AD exhibit high 5-year fatality rates of 35.9%, especially in females (66.7%). For patients with AMI and AD, surgical management tends to have lower mortality.

The pathophysiology of AD is diverse and affected by histopathology and genetic components, (14) which usually results from a tear in the aortic intima. The blood typically propagates rapidly along the length of the aorta and often compromises branch vessels along its path and/or disrupts aortic valve function.

Occasionally, dissection and myocardial infarction may occur concomitantly.

For one reason, AMI may happen when the dissection flap involves the coronary. Iatrogenic acute aortic dissection can also occur during percutaneous coronary intervention. For other reasons, the two diseases just happen on the same patient occasionally. Though the ratio of involvement of LAD and LCx were higher in type B dissection patients compared with type A dissection patients in our results, the indeed needs for surgical intervention were similar between two groups, and both for open surgery or PCI.

In the present study, the overall in-hospital mortality was 10.3% which was relatively low compared with those in the previous studies (1). One reason may result from the improvement of diagnostic equipment and surgical techniques in recent years. Another reason might be that some patients died in the emergency department before a confirmative diagnosis was made. In the IRAD registry, a history of hypertension, which is considered the most common predisposing factor for aortic dissection, was present in more than 70% of patients (20). This was consistent with our study for the most prevalent vascular risk factors were hypertension (69.2%) and current smoker (64.1%). Despite 79.5% of patients being on antihypertensive medication, the control of blood pressure (BP) was poor. Improved primary prevention, in particular, more aggressive management of hypertension and smoking cessation, may reduce future incidence rates, but treatment of resistant or refractory hypertension is likely to remain a challenge.

The Hcy is higher in type B dissection patients compared with type A dissection patients showed in this manuscript, that is consistent with higher incidence of hypertension in type B group in present study. A random clinical trial enrolled 20702 adults confirmed that almost 75% hypertensive patients have elevated Hcy in Chinese population (21, 22), therefore, we thought the higher level of Hcy in type B dissection may result from the higher incidence of hypertension. In addition, Sassi and his colleagues found that Hcy was significantly higher in men than in women (23), that is consistent with our results.

This study has limitations. First, this was a retrospective cohort study, which may underestimate incidence and case fatality by the incomplete inclusion of deaths before hospital admission, which might also bias the assessment of risk factors and predictors of outcome. Second, although this study was the first to fulfill a substantial follow-up of a large series of patients with AMI and AD, its sample size was relatively small in comparison with that of many studies regarding AMI or AD only. Because AMI occurring in patients with AD is a rare condition, it seems impractical for a single center to enroll a large study population with both AMI and HCM. Multicenter studies enrolling much larger study populations are necessary to validate our findings.

## Conclusion

Patients with AMI and AD exhibit high 5-year fatality rates. For these patients, surgical management tends to have lower mortality. Improved management of hypertension and smoking may reduce future incidence rates.

## Data Availability Statement

The raw data supporting the conclusions of this article will be made available by the authors, without undue reservation.

## Ethics Statement

The studies involving human participants were reviewed and approved by Beijing Anzhen Hospital Ethics Committee. Written informed consent for participation was not required for this study in accordance with the national legislation and the institutional requirements.

## Author Contributions

FL and S-CQ contributed to the conception or design of the work. FL, S-CQ, X-CY, and M-LC contributed to the acquisition, analysis, or interpretation of data for the work. FL drafted the manuscript. X-CY and M-LC critically revised the manuscript. All authors gave final approval and agreed to be accountable for all aspects of the work ensuring integrity and accuracy. All authors take responsibility for all aspects of the reliability and freedom from bias of the data presented and their discussed interpretation. All authors contributed to the article and approved the submitted version.

## Funding

This study was supported by grants from the Chaoyang Hospital (CHPY202051).

## Conflict of Interest

The authors declare that the research was conducted in the absence of any commercial or financial relationships that could be construed as a potential conflict of interest. The reviewer ZWZ declared a shared affiliation with one of the authors, FL, to the handling editor.

## Publisher's Note

All claims expressed in this article are solely those of the authors and do not necessarily represent those of their affiliated organizations, or those of the publisher, the editors and the reviewers. Any product that may be evaluated in this article, or claim that may be made by its manufacturer, is not guaranteed or endorsed by the publisher.

## Supplementary Material

Supplementary Table 1 Management and outcomes of acute aotric dissection.

## Abbreviations

AAD: Acute aortic dissection
ACS: acute coronary syndrome
AMI: acute myocardial infarction
CABG: coronary artery bypass grafting
CI: confidence interval
HRs: hazard ratios
PCI: percutaneous coronary intervention.
