# Supplementary material for: Incidence and Outcome of Acute Myocardial Infarction in Patients With Aortic Dissection and Risk Factor Control
Source: Front Surg. 2021 Sep 9;8:678806. doi: 10.3389/fsurg.2021.678806 (PMC8459711; doi:10.3389/fsurg.2021.678806)
Supplement: Supplementary Table 1 — Management and outcomes of acute aotric dissection. [file Data_Sheet_1.docx]

| **Supplemental Table. Management and outcomes of acute aotric dissection** | | | | | |  |  |  |
| --- | --- | --- | --- | --- | --- | --- | --- | --- |
|  | **Male (n = 33) management, No.(%)** | | **Female (n = 6) management, No.(%)** | | **Type A (n = 31) management, No.(%)** | | **Type B (n = 8) management, No.(%)** | |
|  | Surgical | Medical | Surgical | Medical | Surgical | Medical | Surgical | Medical |
| No. , n (%) | 22 (66.7) | 11 (33.3) | 4 (66.7) | 2 (33.3) | 20 (64.5) | 11 (35.5) | 6 (75.0) | 2 (25.0) |
| In-hospital mortality, n (%) | 2 (9.1) | 0 (0.0) | 2 (50.0) | 0 (0.0) | 3 (15.0) | 0 (0.0) | 1 (16.7) | 0 (0.0) |
| Out-hospital mortality, n (%) | 4 (18.2) | 6 (54.5) | 3 (75.0) | 1 (50.0) | 6 (30.0) | 6 (54.5) | 1 (16.7) | 1 (50.0) |
